# Author Correction: Microglial colonization of the developing mouse brain is controlled by both microglial and neural CSF-1

**DOI:** 10.1038/s44318-026-00717-z

**Published:** 2026-02-13

**Authors:** Cécile Bridlance, Sarah Viguier, Nicolas Olivié, Edmond Dupont, Dorine Thobois, Benjamin Mathieu, Jean X Jiang, Guillermina López-Bendito, Melanie Greter, Burkhard Becher, Florent Ginhoux, Aymeric Silvin, Esther Klingler, Sonia Garel, Morgane Sonia Thion

**Affiliations:** 1 Centre Interdisciplinaire de Recherche en Biologie (CIRB), Collège de France, Université PSL, CNRS, INSERM, 75005 Paris, France; 2 Institut de Biologie de l’École Normale Supérieure (IBENS), École Normale Supérieure, CNRS, INSERM, Université PSL, 75005 Paris, France; 3 Sorbonne Université, Collège Doctoral, F-75005 Paris, France; 4 Department of Biochemistry and Structural Biology, University of Texas Health Science Center, San Antonio, TX 78229-3900 USA; 5 Instituto de Neurociencias de Alicante, Universidad Miguel Hernández-Consejo Superior de Investigaciones Científicas (UMH-CSIC), San Juan de Alicante, Alicante, Spain; 6 Institute of Experimental Immunology, University of Zurich, Zurich, Switzerland; 7 INSERM U1015, Gustave Roussy Cancer Campus, Villejuif, 94800 France; 8 Singapore Immunology Network (SIgN), Agency for Science, Technology and Research, Singapore, 138648 Singapore; 9 VIB-KU Leuven Center for Brain & Disease Research, 3000 Leuven, Belgium; 10 KU Leuven, Department of Neurosciences, Leuven Brain Institute, 3000 Leuven, Belgium; 11 Collège de France, Université PSL, 75005 Paris, France

## Abstract

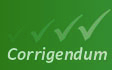

**Correction to:** *The EMBO Journal* (2026) 45:151–181. 10.1038/s44318-025-00625-8 | Published online 17 November 2025

**Author's name is corrected**.

The 13th author’s name is misspelled.

Esther Klinger

Is corrected to:

**Esther Klingler**

