# Supplementary material for: Microglial colonization of the developing mouse brain is controlled by both microglial and neural CSF-1
Source: EMBO J. 2025 Nov 17;45(1):151–81. doi: 10.1038/s44318-025-00625-8 (PMC12759073; doi:10.1038/s44318-025-00625-8)
Supplement: Supplementary file 1 — Appendix [file 44318_2025_625_MOESM1_ESM.pdf]

Appendix for:

Microglial colonization of the developing mouse brain is controlled by both microglial and neural CSF1

Bridlance *et al.*

Serial no.	Table of contents	Page number
1	Appendix Figure S1	1
2	Appendix Figure S2	3
3	Appendix Figure S3	5

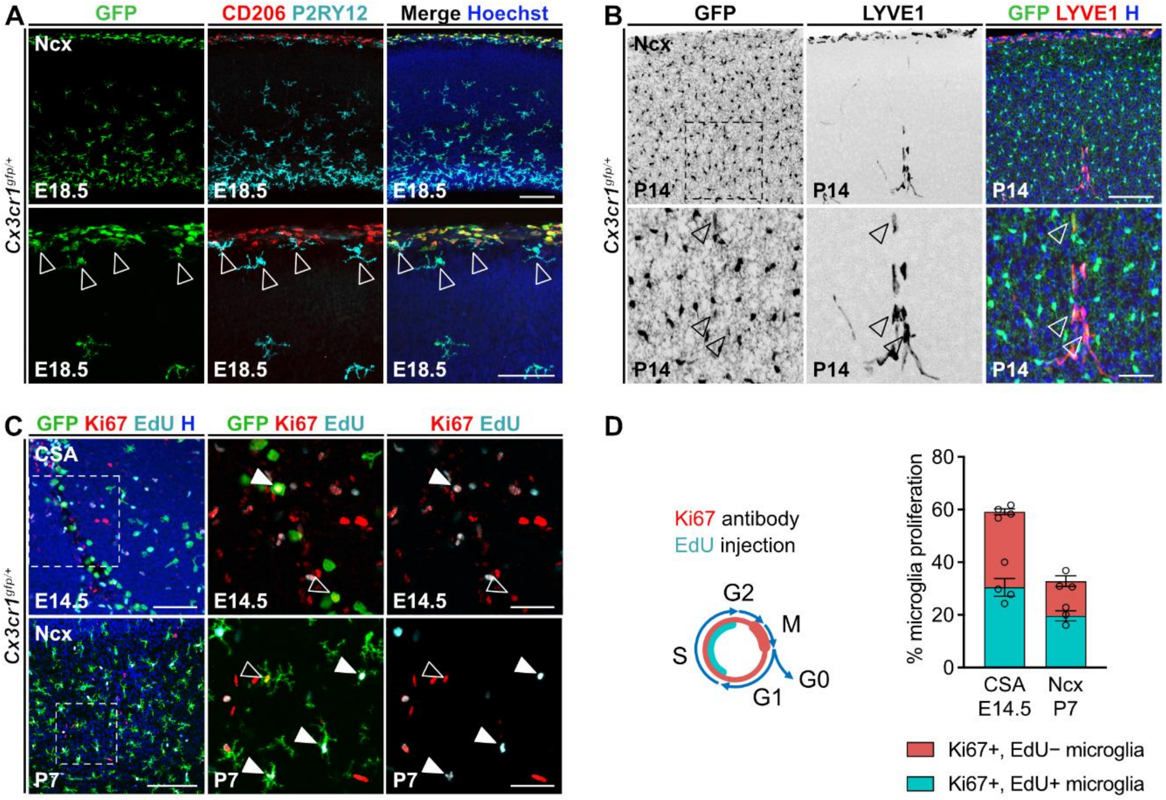

Appendix Figure S1. Identification of microglia, BAMs and proliferative microglia

(A) Immunolabeling of coronal brain sections from E18.5 *Cx3cr1*$^{gfp/+}$ embryos showing co-expression of GFP (labelling microglia and BAMs) with CD206 (labelling BAMs) or P2RY12 (labelling microglia) in the somatosensory cortex. Microglia in the marginal zone (open arrowheads) and BAMs in the overlaying meninges can be further distinguished by their respective ramified and round morphologies.

(B) Immunolabeling of coronal brain sections from P14 *Cx3cr1*$^{gfp/+}$ mice showing GFP and LYVE1 co-labeling of perivascular macrophages (open arrowheads) in the somatosensory cortex. They are found in the perivascular space along blood vessels, which are visible as a Hoechst-intense line, and display a low GFP intensity and elongated morphology that distinguished them from microglia.

(C) Immunolabeling of coronal brain sections from E14.5 and P7 *Cx3cr1*$^{gfp/+}$ mice showing expression of GFP, Ki67 and EdU at the CSA (top) and EDWM (bottom). Virtually all EdU positive cells were also Ki67-positive (solid arrowheads).

(D) Quantification of the proportion of proliferative microglia (Ki67-positive or EdU positive) within the CSA at E14.5 (n=4 from one litter) or in the neocortex at P7 (n=3 from one litter) in *Cx3cr1*$^{gfp/+}$ mice. Roughly half of Ki67-positive microglia were also

EdU-positive, consistent with the fact that Ki67 labels all phases of the cell cycle whereas EdU only targets cells in S-phase.

Data are presented as mean ± SEM.

Scale bars: 150µm (A, low mag) and (B, low mag); 100µm (A, high mag) and (C, low mag); 50µm (B, high mag) and (C, high mag).

CSA, cortico-striatal-amygdalar boundary; H, Hoechst; Ncx, neocortex.

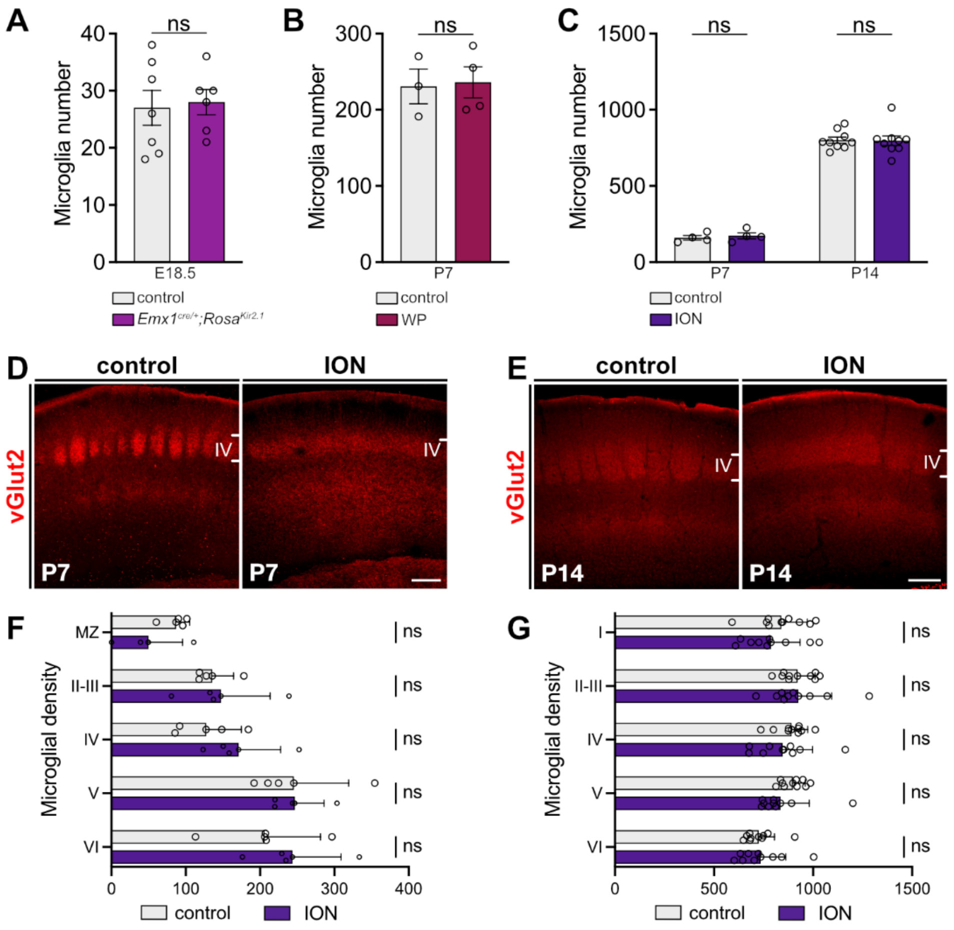

Appendix Figure S2. Infraorbital nerve section does not impact cortical microglial numbers or distribution

(A) Microglial numbers (IBA1-positive cells) in associated control and *Emx1$^{cre/+}$;Kir2.1* (mutant) mice at E18.5 ($n_{control}$=7; n_{mutant}=6; from two distinct litters).

(B) Microglial numbers (IBA1-positive cells) in associated control mice and mice having undergone WP at P7 ($n_{control}$=4; n_{WP}=3; from one litter).

(C) Microglial numbers (IBA1-positive cells) in control mice and mice having undergone ION at P7 (littermate control, n=4 from two distinct litters) and P14 (littermate control, n=9 from three distinct litters).

(D) vGlut2 immunolabeling of coronal brain sections from P7 control mice and mice having undergone ION showing the presence (controls) or absence (ION) of barrels in the somatosensory cortex (controls are the cortex of the ipsilateral hemisphere and ION of the contralateral hemisphere).

(E) vGlut2 immunolabeling of coronal brain sections from P14 control and ION mice showing barrels in the somatosensory cortex.

(F) Microglial density (IBA1-positive cells/mm^2) across cortical layers at P7 in control and ION mice performed at P1 (littermate control, n=4; from two distinct litters).
(G) Microglial density (IBA1-positive cells/mm^2) across cortical layers at P14 in control and ION mice performed at P1 (littermate control, n=9; from three distinct litters).
Data are presented as mean ± SEM. Two-way ANOVA with Sidak's post hoc test were performed to assess differences (B and F). ns, not significant.
Scale bars: 200µm (A) and 250µm (B).
ION, infraorbital nerve section; WP, whisker plucking; I, II, III, IV, V and VI are respectively cortical layers I, II, III, IV, V and VI.

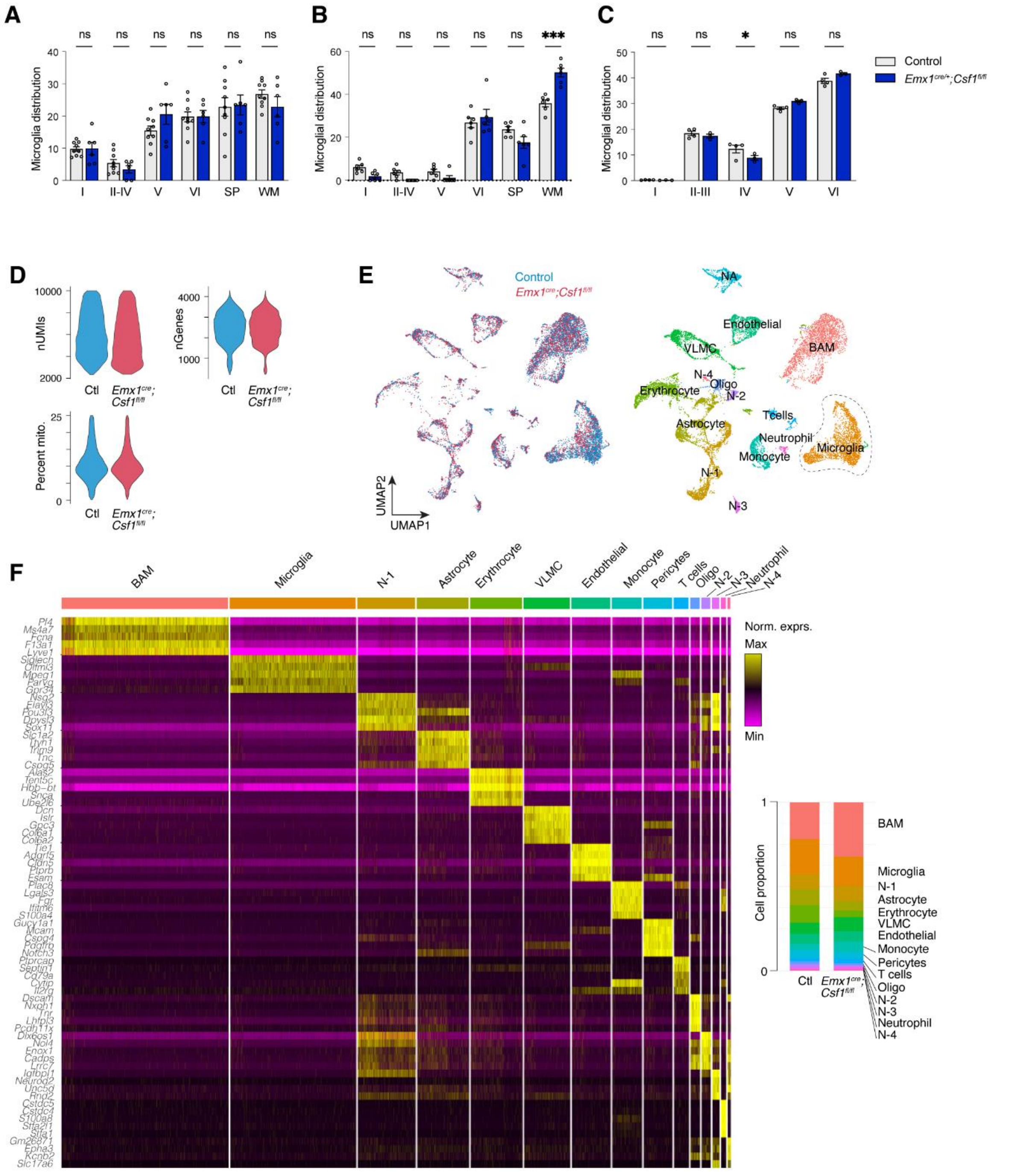

Appendix Figure S3. Single cell RNA-sequencing of microglia-enriched cortex samples from control and *Emx1^{Cre};Csf1$^{fl/fl}$* P3 pups

(A) Microglial distribution (% of IBA1-positive cells/mm² per layer) in the different layers of the neocortex of P0 control and *Emx1$^{cre/+}$;Csf1$^{fl/fl}$* pups ($n_{control}$=10, n_{mutant}=5; from two distinct litters).

(B) Microglial distribution (% of IBA1-positive cells/mm² per layer) in the different layers of the neocortex of P3 control and *Emx1$^{cre/+}$;Csf1$^{fl/fl}$* pups ($n_{control}$=6, n_{mutant}=6; from two distinct litters per stage)(P_{WM} < 0.0001).

(C) Microglial distribution (% of IBA1-positive cells/mm² per layer) in the different layers of the neocortex of P7 control and *Emx1$^{cre/+}$;Csf1$^{fl/fl}$* pups ($n_{control}$=4, n_{mutant}=3; from two distinct litters per stage)(P_{IV} = 0.0429).

(D) Violin plots of the number of unique molecular identifiers (UMIs) and genes, and of the percentage of mitochondrial (mito.) RNA detected per cell used for the analysis (n = 12,287 control cells; n = 7459 *Emx1^{Cre}; Csf1$^{fl/fl}$* cells).

(E) Quality-controlled cells projected in the Uniform Manifold Approximation and Projection (UMAP) space labeled by condition (left) and cell type (right).

(F) Top marker genes per cell type allowing cell type annotation (left) and proportion of cells per cell type across conditions (right).

Data are presented as mean ± SEM. Two-way ANOVA with Sidak's post hoc test were performed to assess differences (A-C). ns, not significant, * $P < 0.05$, *** $P < 0.001$.

Ctl, control; BAM, border associated macrophage; mito, mitonchondrial; NA, non-annotated; N, neurons; Oligo, oligodendrocyte; SP, subplate; UMIs, unique molecular identifiers; VLMC, Vascular and Leptomeningeal Cells; WM, white matter. I, II, III, IV, V and VI are respectively cortical layers I, II, III, IV, V and VI.
