# Supplementary figures and images for: Microglial colonization of the developing mouse brain is controlled by both microglial and neural CSF-1
Source: EMBO J. 2025 Nov 17;45(1):151–81. doi: 10.1038/s44318-025-00625-8 (PMC12759073; doi:10.1038/s44318-025-00625-8)

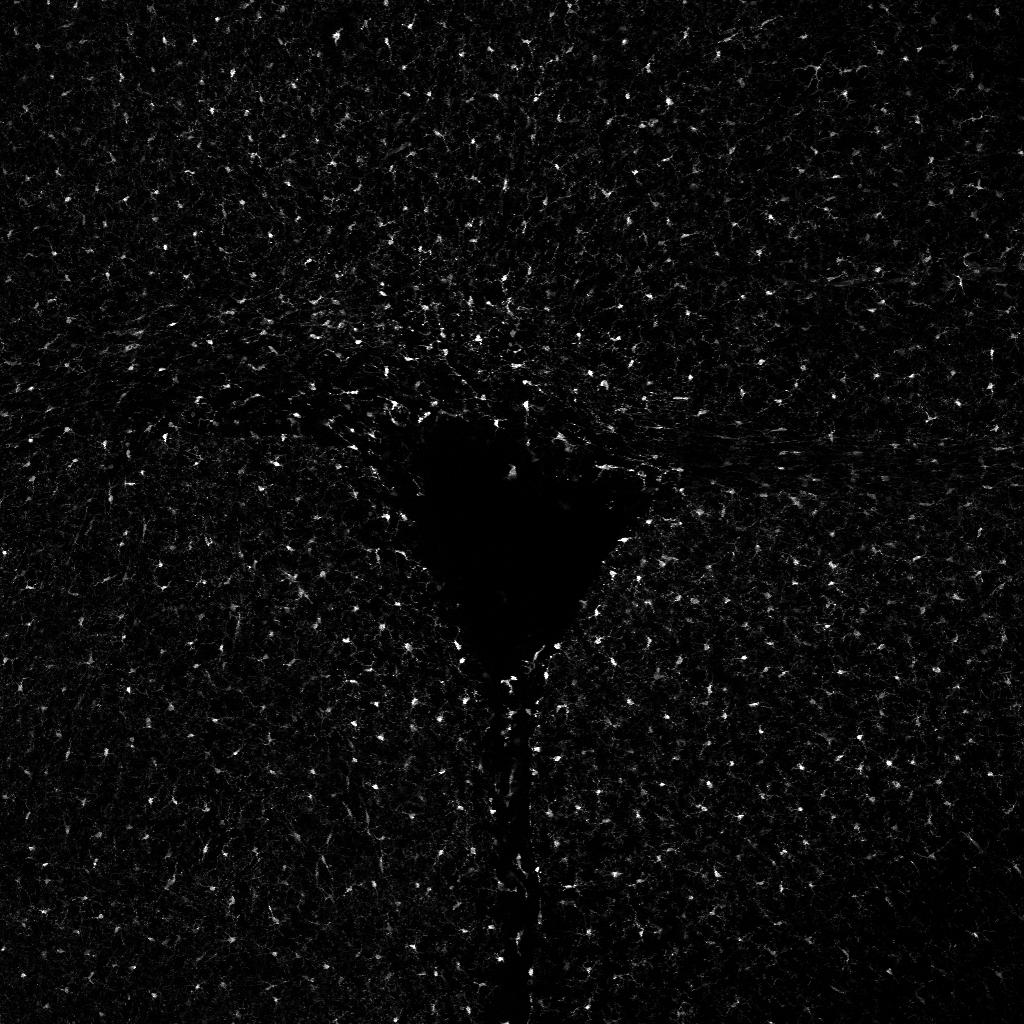

Supplement: Supplementary file 4 — Source data Fig. 1 [file 44318_2025_625_MOESM4_ESM.zip › SD figure 1/1J/1J EDWM P14.tif]

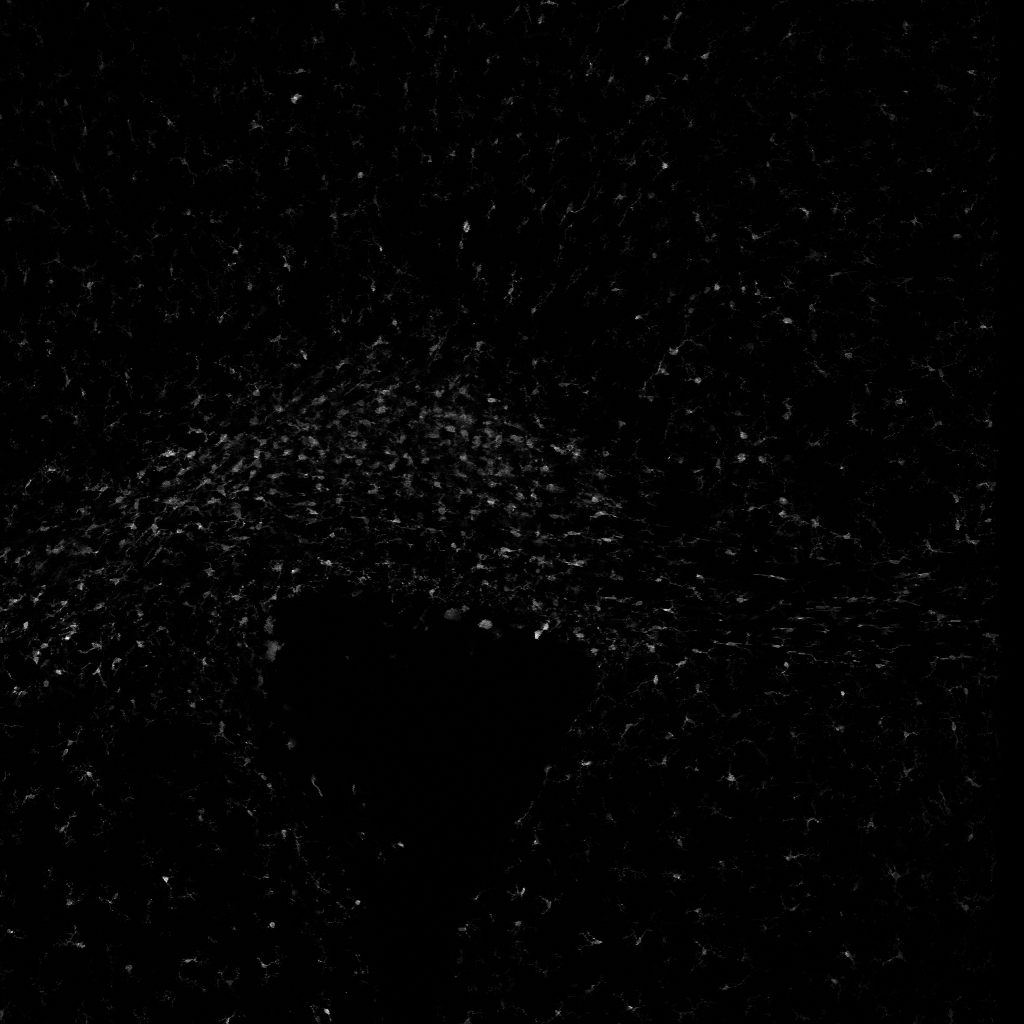

Supplement: Supplementary file 4 — Source data Fig. 1 [file 44318_2025_625_MOESM4_ESM.zip › SD figure 1/1J/1J EDWM P9.tif]

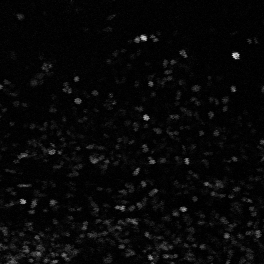

Supplement: Supplementary file 4 — Source data Fig. 1 [file 44318_2025_625_MOESM4_ESM.zip › SD figure 1/1M/1M high mag.tif]

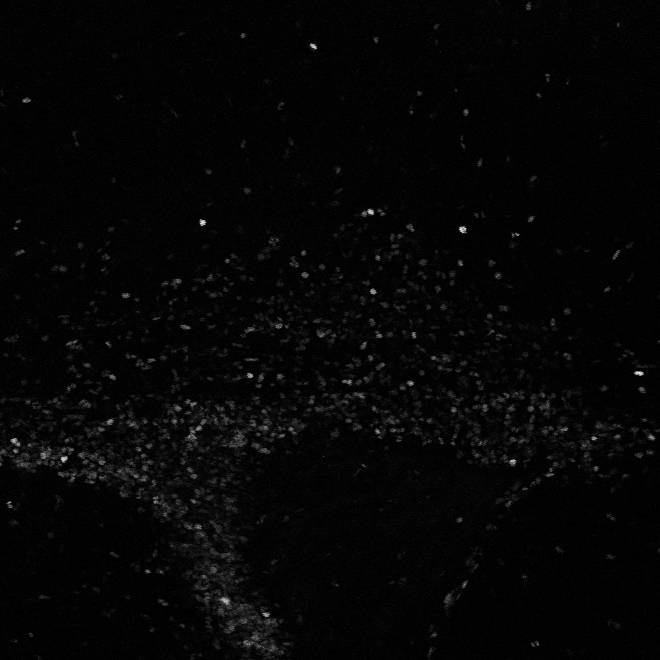

Supplement: Supplementary file 4 — Source data Fig. 1 [file 44318_2025_625_MOESM4_ESM.zip › SD figure 1/1M/1M.tif]

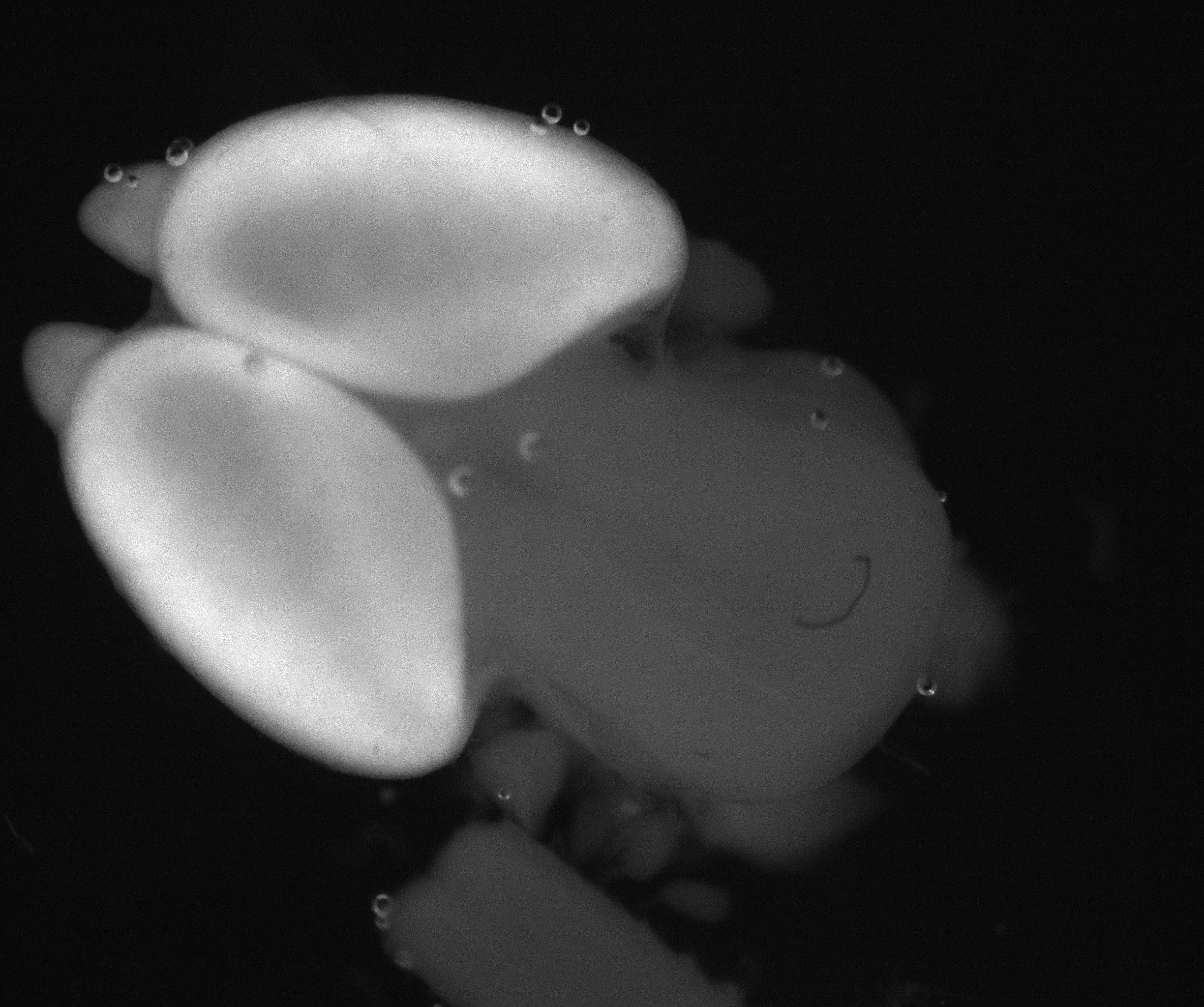

Supplement: Supplementary file 6 — Source data Fig. 3 [file 44318_2025_625_MOESM6_ESM.zip › SD figure 3/3B/3B 14.5.tif]

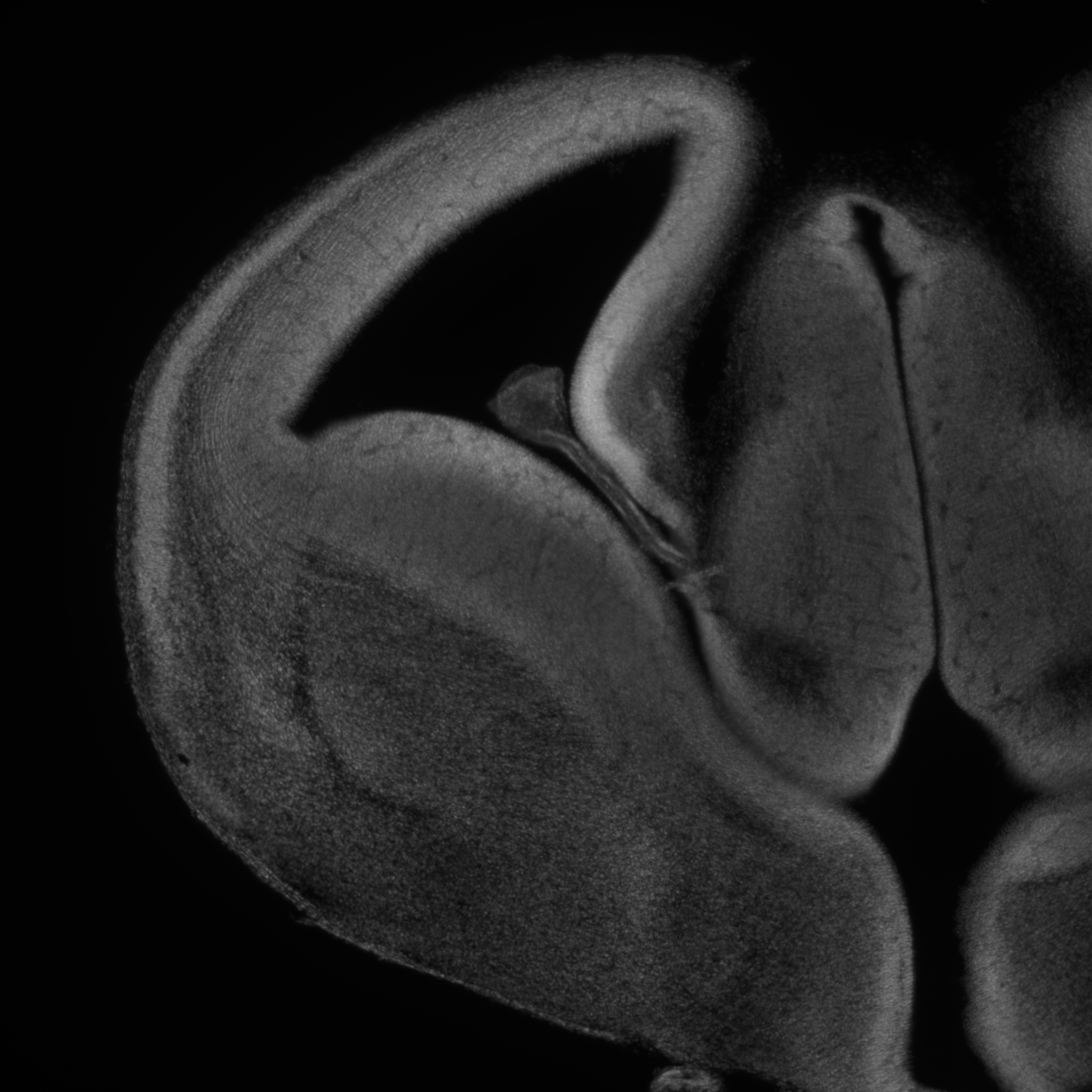

Supplement: Supplementary file 6 — Source data Fig. 3 [file 44318_2025_625_MOESM6_ESM.zip › SD figure 3/3C/3C control.tif]

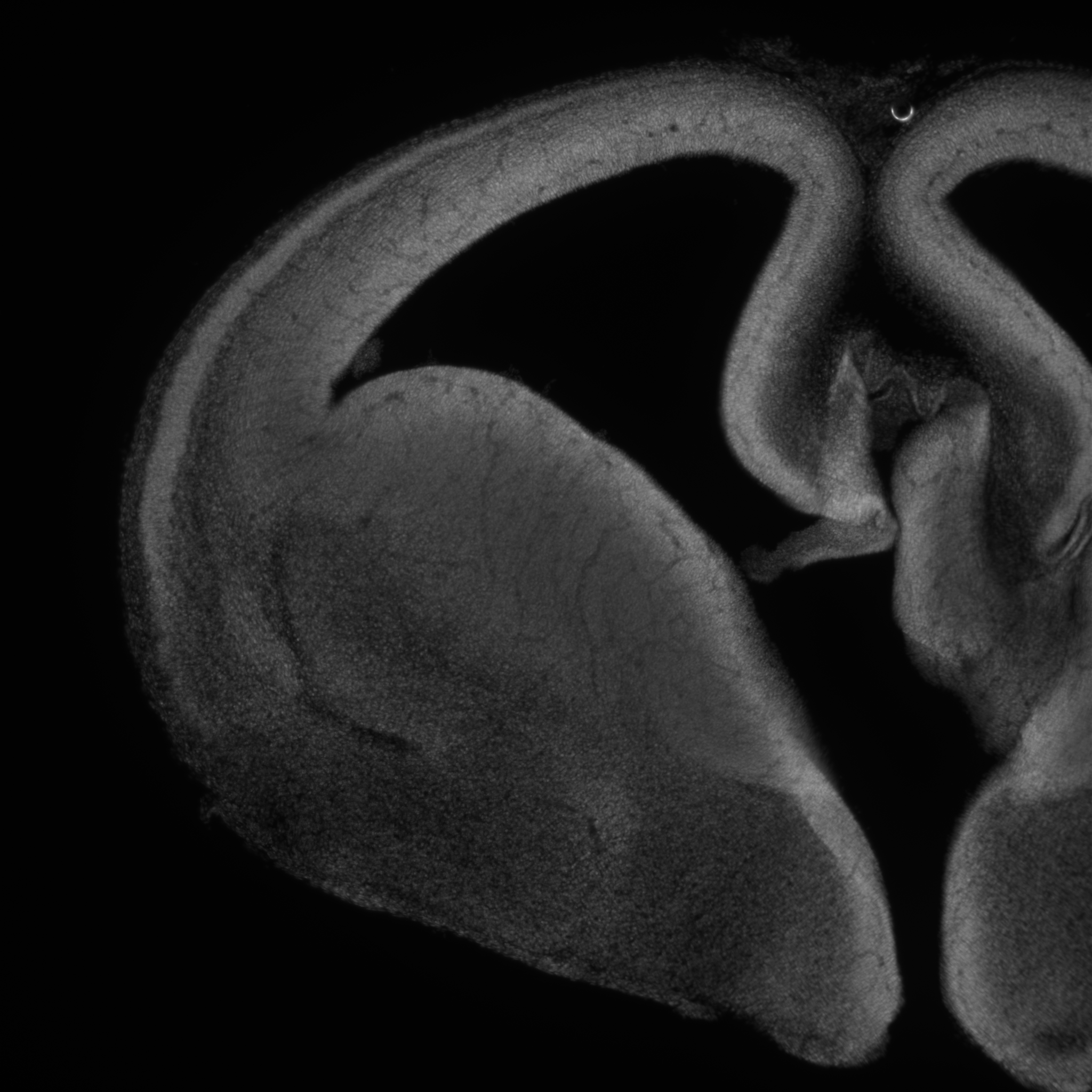

Supplement: Supplementary file 6 — Source data Fig. 3 [file 44318_2025_625_MOESM6_ESM.zip › SD figure 3/3C/3C mutant.tif]

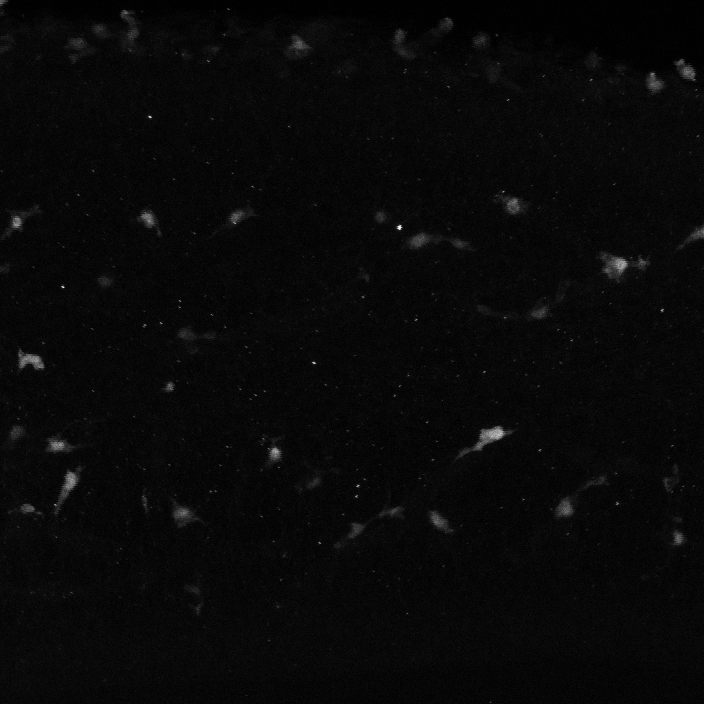

Supplement: Supplementary file 6 — Source data Fig. 3 [file 44318_2025_625_MOESM6_ESM.zip › SD figure 3/3E/3E E14.5 control.tif]

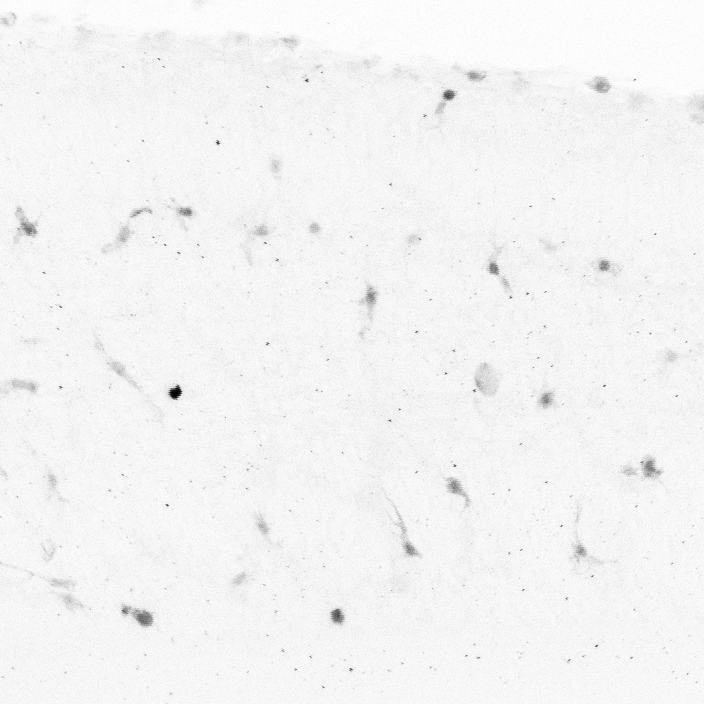

Supplement: Supplementary file 6 — Source data Fig. 3 [file 44318_2025_625_MOESM6_ESM.zip › SD figure 3/3G/3G control zoom.tif]

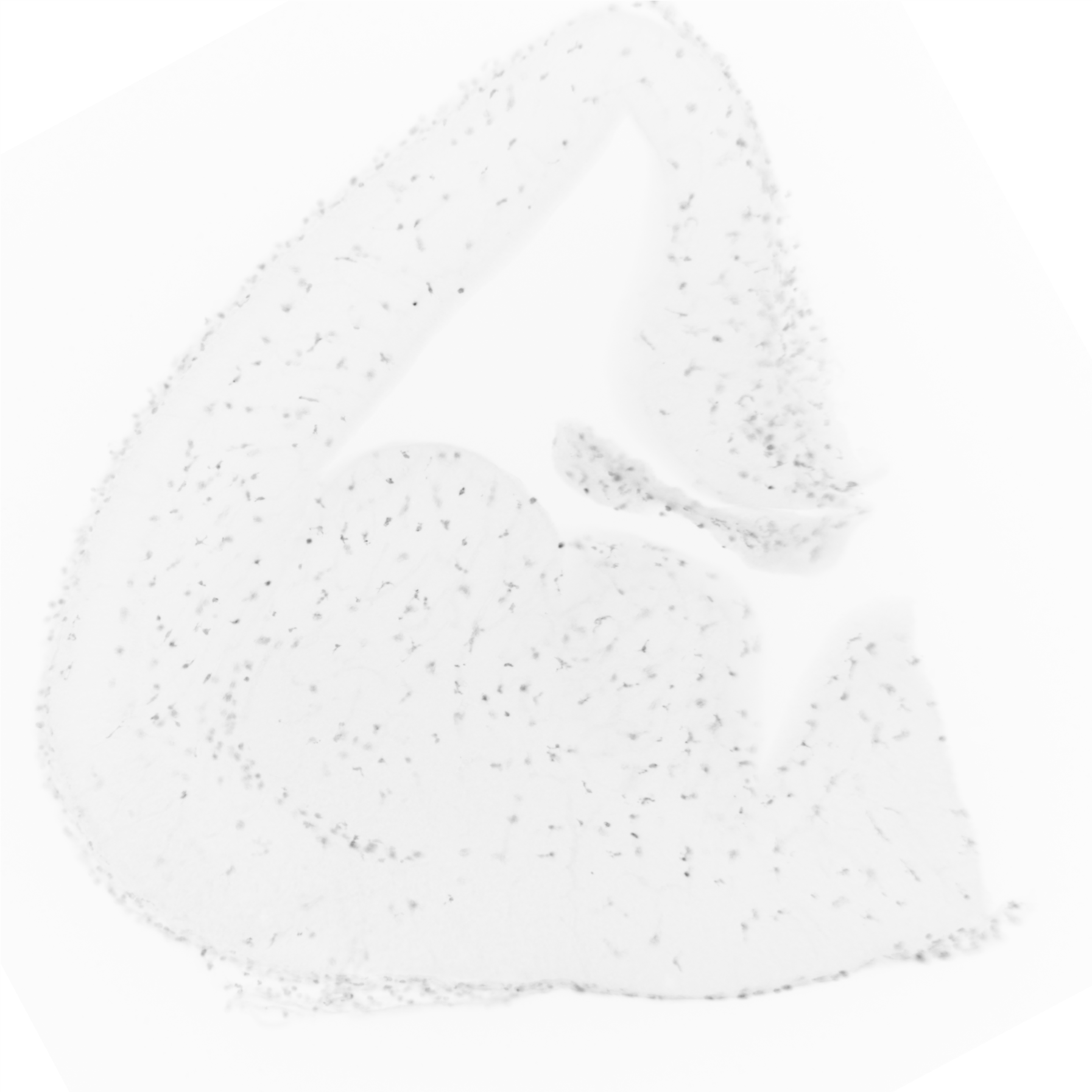

Supplement: Supplementary file 6 — Source data Fig. 3 [file 44318_2025_625_MOESM6_ESM.zip › SD figure 3/3G/3G control.tif]

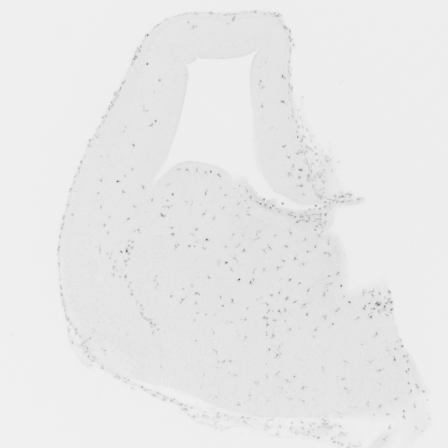

Supplement: Supplementary file 6 — Source data Fig. 3 [file 44318_2025_625_MOESM6_ESM.zip › SD figure 3/3G/3G het.tif]

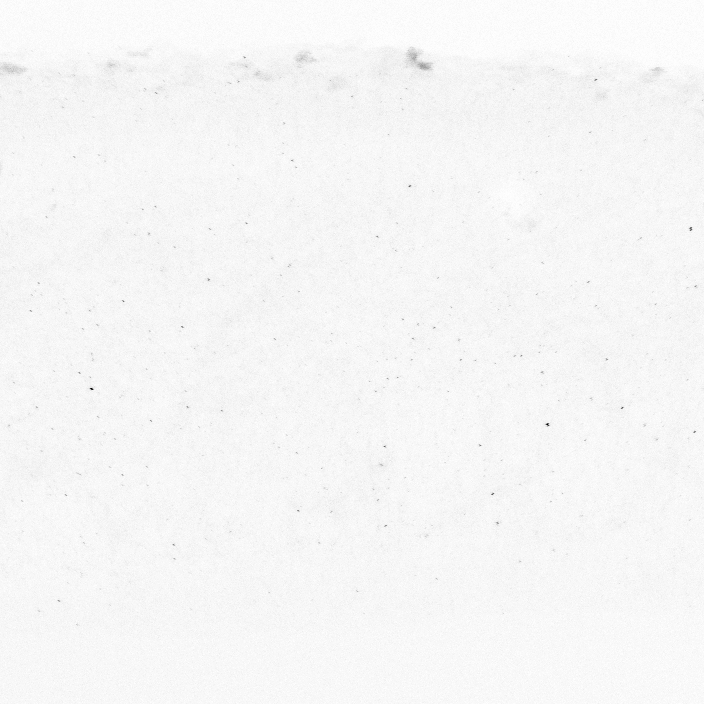

Supplement: Supplementary file 6 — Source data Fig. 3 [file 44318_2025_625_MOESM6_ESM.zip › SD figure 3/3G/3G mutant zoom.tif]

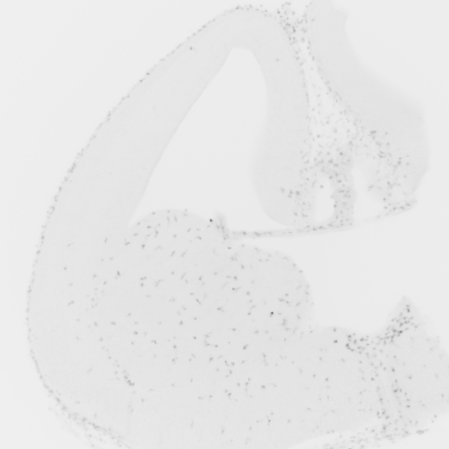

Supplement: Supplementary file 6 — Source data Fig. 3 [file 44318_2025_625_MOESM6_ESM.zip › SD figure 3/3G/3G mutant.tif]

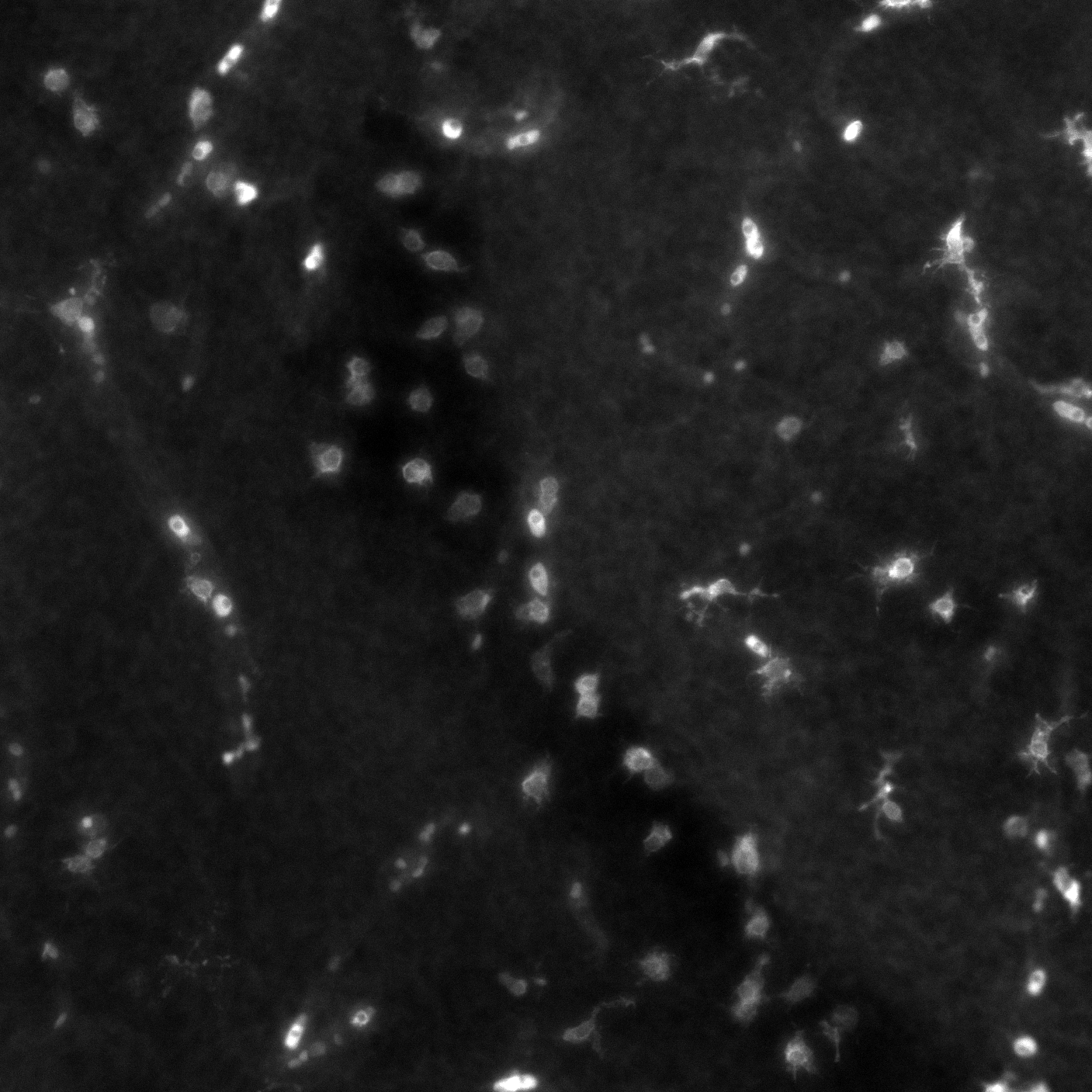

Supplement: Supplementary file 9 — Source data Fig. 6 [file 44318_2025_625_MOESM9_ESM.zip › SD figure 6/6B/6B control.tif]

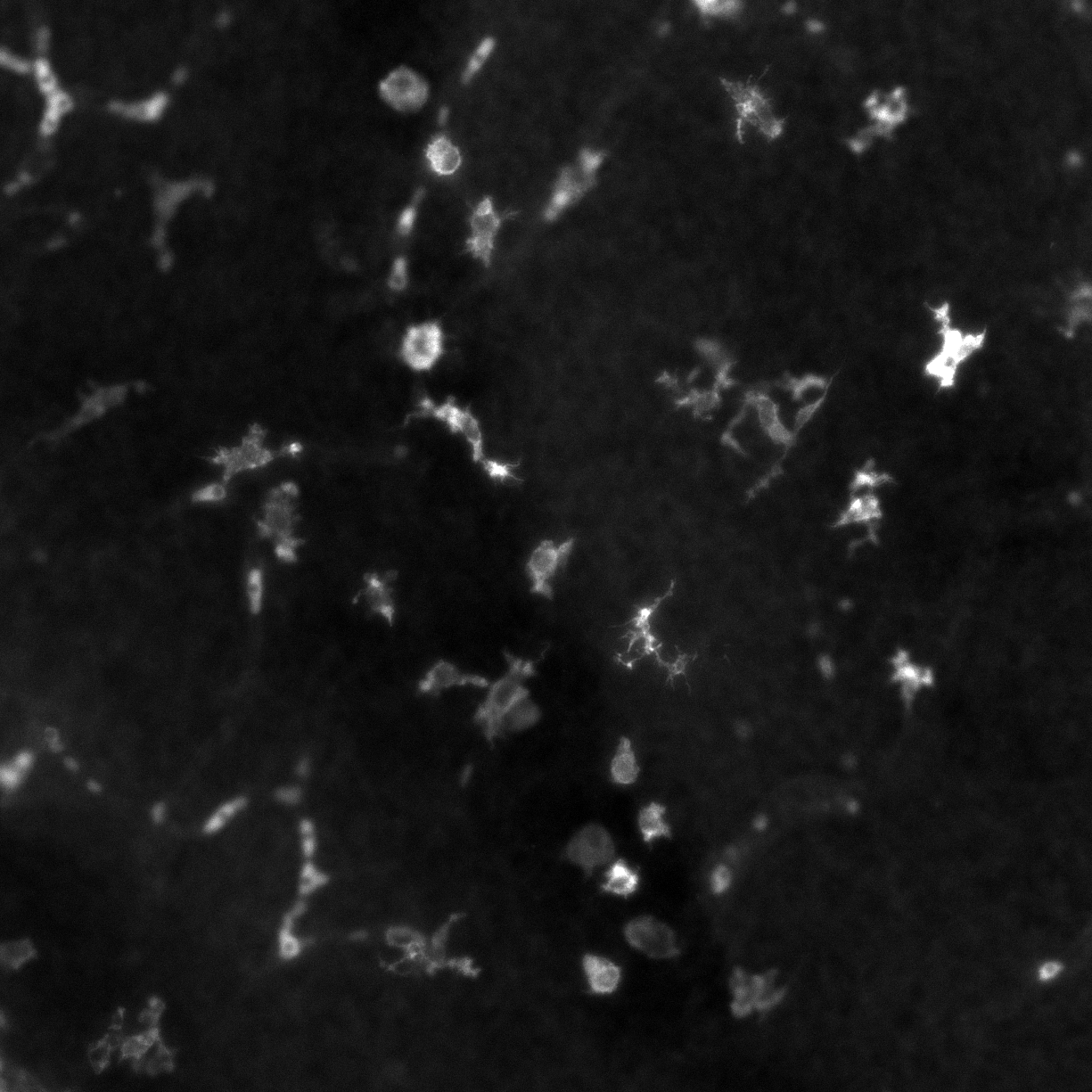

Supplement: Supplementary file 9 — Source data Fig. 6 [file 44318_2025_625_MOESM9_ESM.zip › SD figure 6/6B/6B mutant.tif]

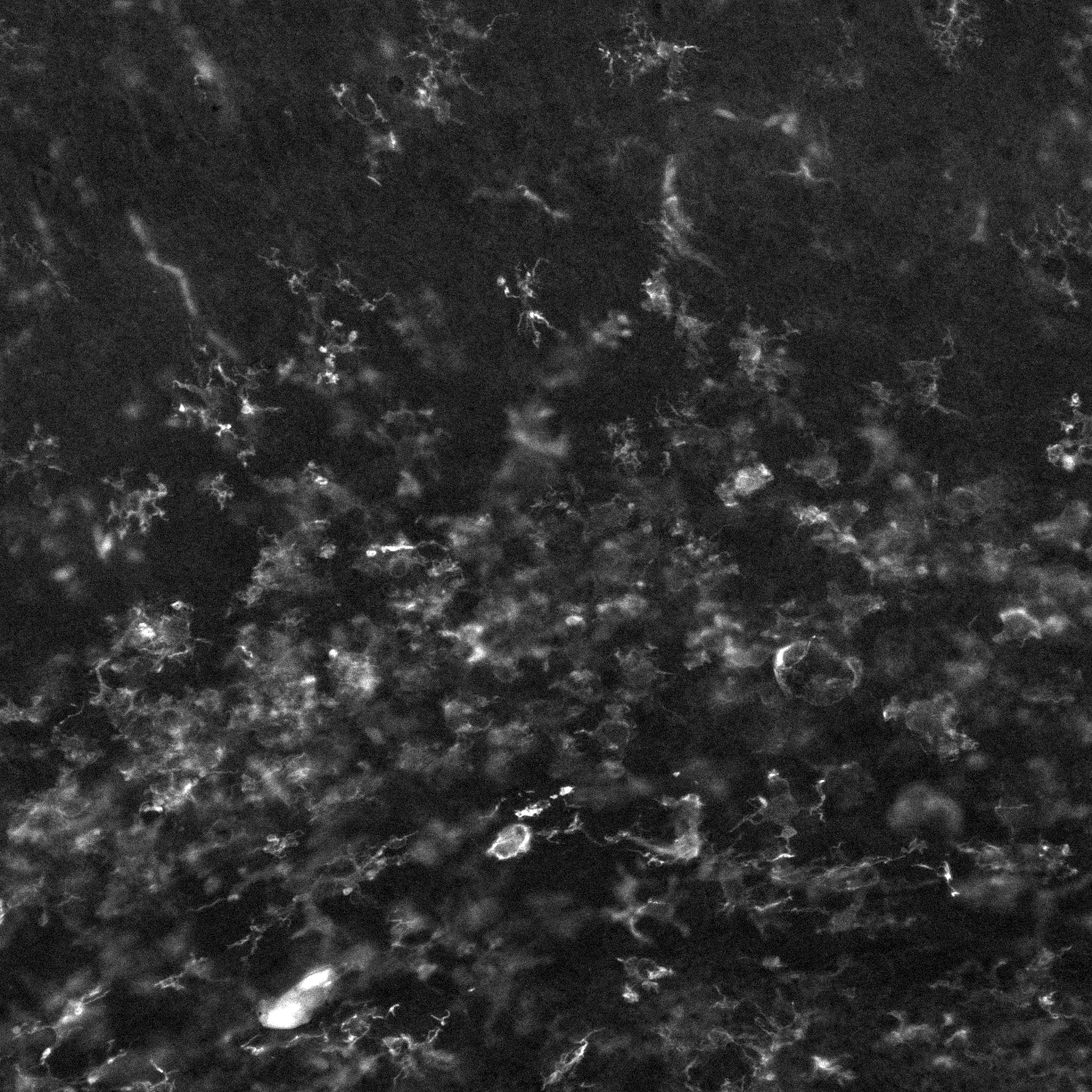

Supplement: Supplementary file 9 — Source data Fig. 6 [file 44318_2025_625_MOESM9_ESM.zip › SD figure 6/6D/6D control.tif]

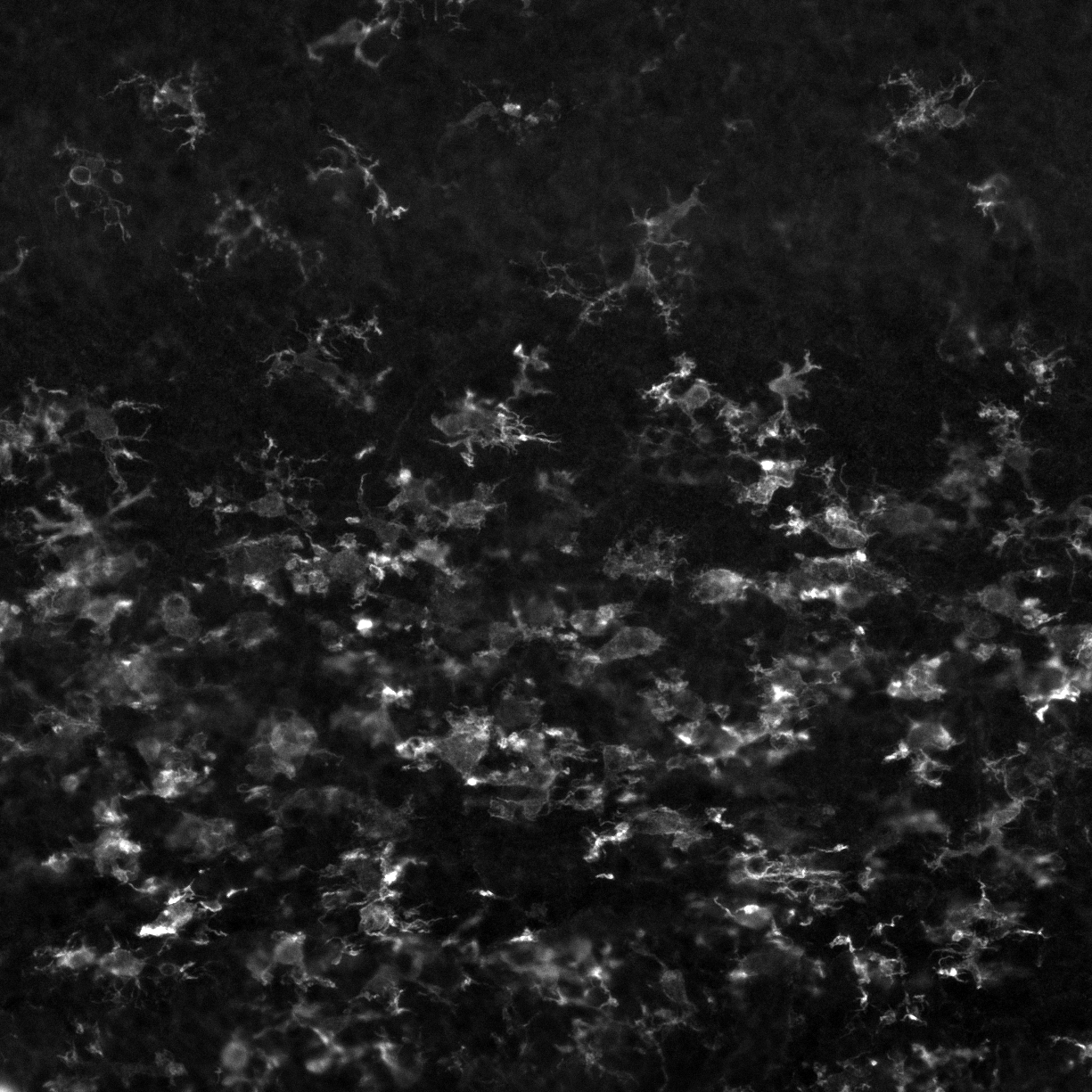

Supplement: Supplementary file 9 — Source data Fig. 6 [file 44318_2025_625_MOESM9_ESM.zip › SD figure 6/6D/6D mutant.tif]
