# Supplementary material for: Microglial colonization of the developing mouse brain is controlled by both microglial and neural CSF-1
Source: EMBO J. 2025 Nov 17;45(1):151–81. doi: 10.1038/s44318-025-00625-8 (PMC12759073; doi:10.1038/s44318-025-00625-8)
Supplement: Supplementary file 11 — Expanded View Figures [file 44318_2025_625_MOESM11_ESM.pdf]

Expanded View Figures

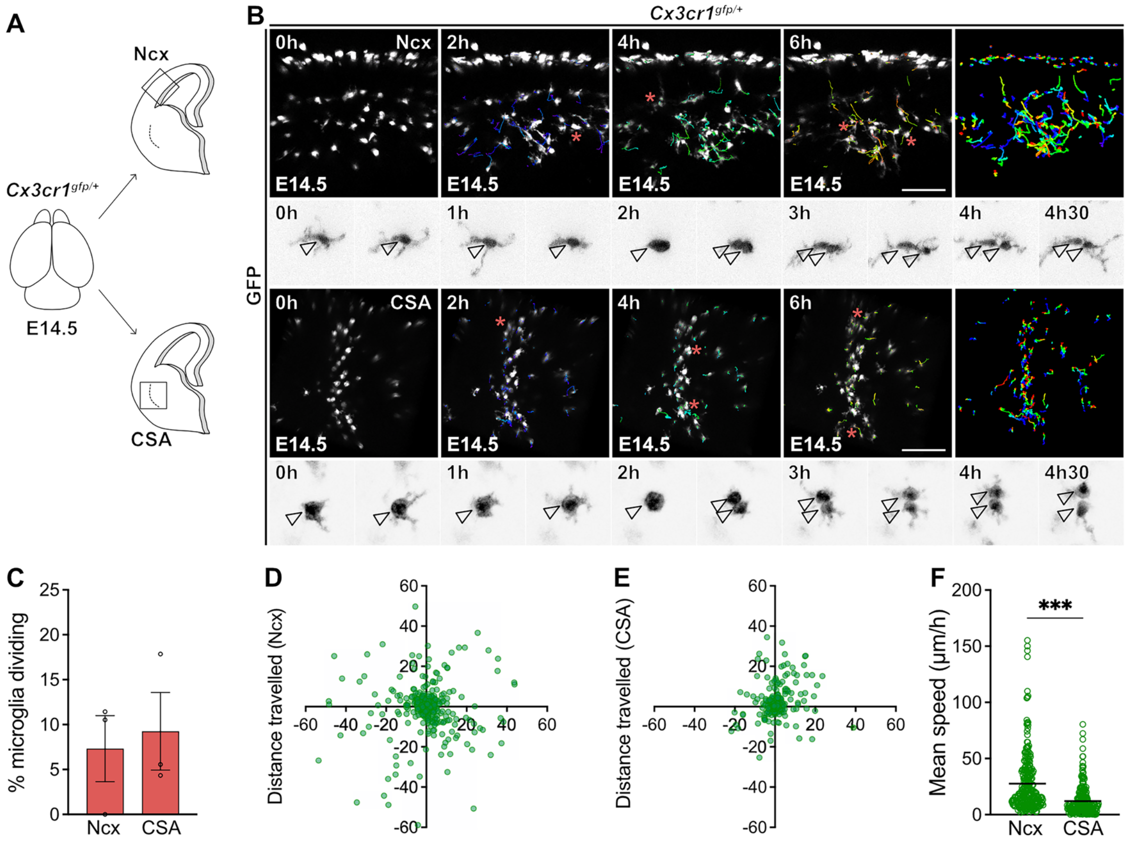

Figure EV1. Different dynamics of microglia in the embryonic neocortex and at the CSA.

(**A**) Schematic representation of a *Cx3cr1*$^{gfp/+}$ E14.5 coronal brain hemisection illustrating the areas where two-photon time-lapse imaging was performed. (**B**) Sample images with superimposed trajectories of individual cells from video tracking of microglia (GFP-positive cells) in E14.5 *Cx3cr1*$^{gfp/+}$ embryos in the neocortex (top) or at the CSA (bottom) (length of recording, 6 h). Colors illustrate the speed of migration and stars highlight dividing microglia. Asterisks indicate single microglia dividing over the course of imaging. High magnification images track single microglia dividing over the course of imaging (open arrowheads). (**C**) Percentage of microglia dividing during the time-course of the recordings. (**D**, **E**) Distance traveled by individual cells tracked during the time-course of the recordings in the neocortex (**D**) or at the CSA (**E**). (**F**) Mean speed of individual cells tracked during the time-course of the recordings in the neocortex or at the CSA ($P < 0.0001$). Three embryos were imaged in each area from six distinct litters. Data were presented as mean ± SEM. Two-sided unpaired Mann–Whitney test (**C**) or Student's *t*-test (**F**) were performed to assess differences ns not significant, $^{***}P < 0.001$. Scale bars: 100 µm. CSA cortico-striato-amygdalar boundary, Ncx neocortex.

 © The Author(s)

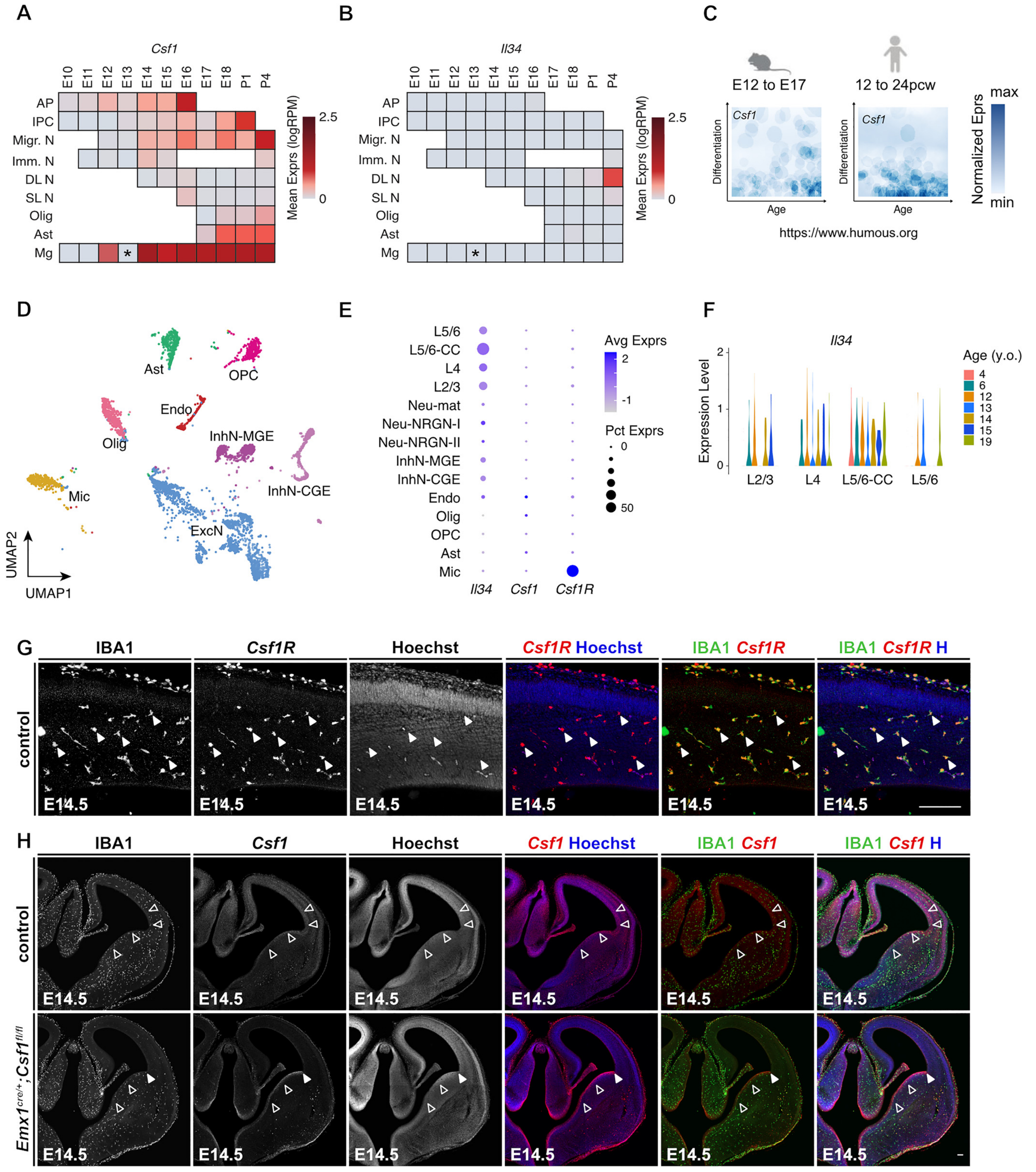
A
Csf1
E10 E11 E12 E13 E14 E15 E16 E17 E18 P1 P4
AP
IPC
Migr. N
Imm. N
DL N
SL N
Olig
Ast
Mg
Mean Exprs (logRPM)
2.5
0
B
Il34
C
E12 to E17
12 to 24pcw
Differentiation
Age
Normalized Eprs
max
min
https://www.humous.org
D
Ast
OPC
Endo
Olig
InhN-MGE
InhN-CGE
Mic
ExcN
UMAP2
UMAP1
E
L5/6
L5/6-CC
L4
L2/3
Neu-mat
Neu-NRGN-I
Neu-NRGN-II
Endo
OPC
Avg Exprs
Pct Exprs
50
Il34
Csf1
Csf1R
F
Expression Level
Age (y.o.)
G
IBA1
Csf1R
Hoechst
Csf1R Hoechst
IBA1 Csf1R
IBA1 Csf1R H
control
E14.5
H
Csf1
Csf1 Hoechst
IBA1 Csf1
IBA1 Csf1 H
Emx1cre/+;Csf1fl/fl

Figure EV2. Spatiotemporal dynamics of *Csf1*, *Il34*, and *Csf1R* expression in the developing mouse and human cortex.

(**A**, **B**) Heatmaps of single-cell gene expression of *Csf1* (**A**) *or Il34* (**B**) in neural cells of the neocortex and in microglia during development (mean reads per million). Data extracted from the available dataset produced by (Di Bella et al, 2021). Squares were left empty when data were not available. At E13 (marked by stars), only five microglial cells were identified. (**C**) Representative cyto-temporal gene expression landscape showing prenatal mouse and human *Csf1* expression in radial glia from HuMous.org (Javed et al, 2025). (**D**) Postnatal cortical human single-nucleus RNA-sequencing data represented in the UMAP space from (Baldassari et al, 2025). Original data were from (Velmeshev et al, 2023)(control individuals). (**E**) Dot plot showing the relative expression levels of *Il34*, *Csf1*, and *Csf1R* in postnatal cortical human cells. The color represents the normalized expression level across all cells within a cluster, while the dot size indicates the percentage of cells expressing each gene in that cluster. (**F**) Postnatal expression of *Il34* in human excitatory neuron types from brains of individuals aged 4 to 19 years. (**G**) RNAscope experiments on coronal brain sections from E14.5 control embryos showing *Csf1r* expression in IBA1-positive microglia in the neocortex (open arrowheads) ($n = 4$ from two litters). (**H**) RNAscope experiments on coronal brain sections from E14.5 control and *Emx1*^{cre};*Csf1*$^{fl/fl}$ embryos showing loss of *Csf1* expression (open arrowheads) in the neocortex of mutant mice (solid arrowhead) ($n_{control} = 5$; $n_{mutant} = 7$ from at least 3 litters). Scale bars: 100 µm (**G**, **H**). AP apical progenitors, Ast astrocytes, CGE caudal ganglionic eminence, DL N deep layer neurons, Imm. N immature neuron, IPC intermediate progenitor cell, MGE medial ganglionic eminence, Migr. N migrating neuron, Neu neuron, NRGN GluN-enriched protein neurogranin, Olig oligodendrocytes, pcw post-conceptional weeks, RPM reads per million, SL N superficial layer neurons.

 © The Author(s)

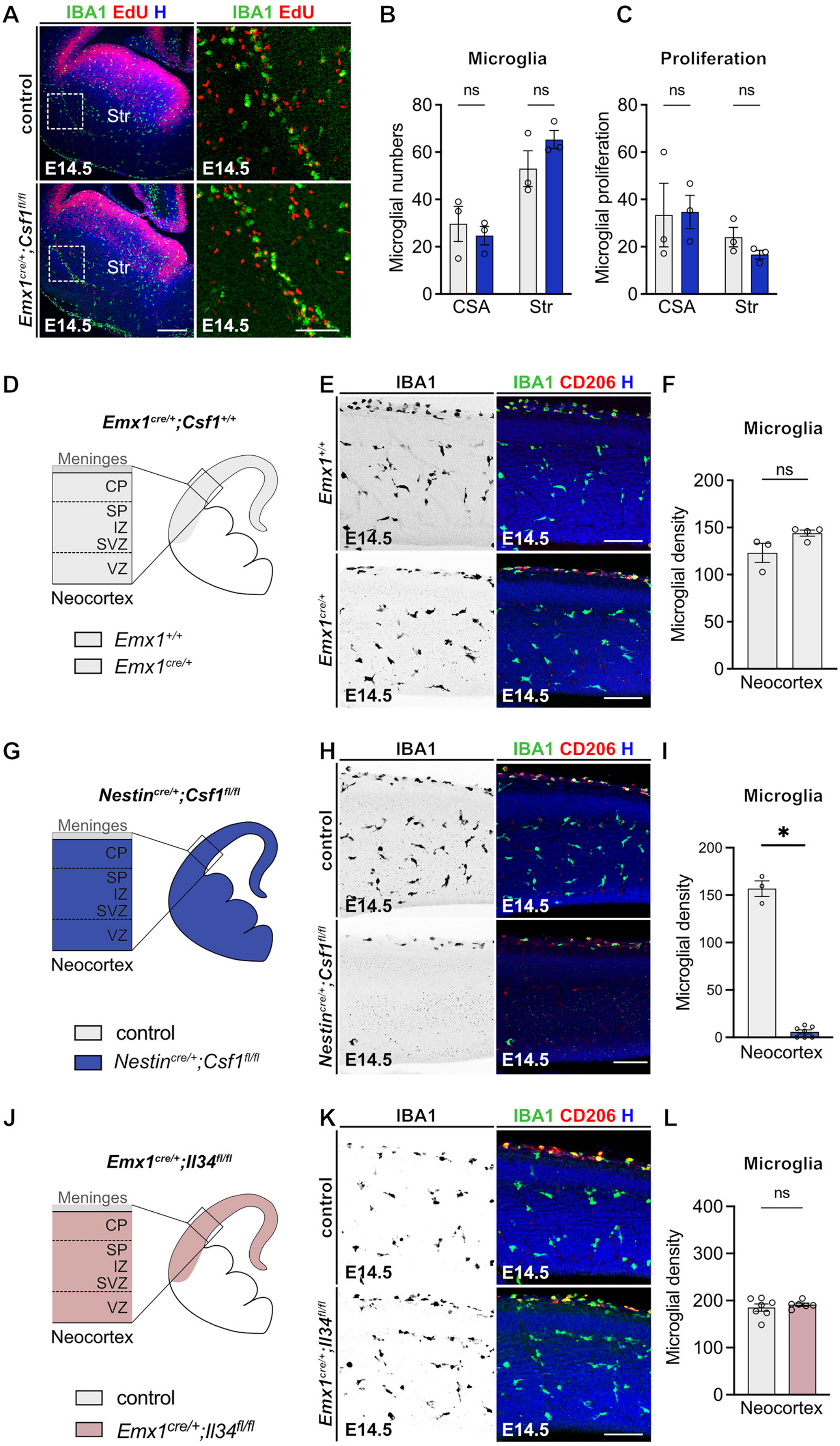
A
IBA1 EdU H
IBA1 EdU
control
Emx1cre/+;Csf1fl/fl
Str
E14.5
B
Microglia
Microglial numbers
ns
CSA
Str
C
Proliferation
Microglial proliferation
D
Emx1cre/+;Csf1+/+
Meninges
CP
SP
IZ
SVZ
VZ
Neocortex
Emx1+/+
Emx1cre/+
E
IBA1
IBA1 CD206 H
F
Microglial density
G
Nestincre/+;Csf1fl/fl
control
H
I
*
J
Emx1cre/+;Il34fl/fl
K
L

Figure EV3. Impacts of *Csf1* and *Il34* on microglial density during early development.

(**A**) Immunolabeling of coronal brain sections from E14.5 control and *Emx1*$^{cre/+}$;*Csf1*$^{fl/fl}$ embryos showing IBA1 and EdU used to identify proliferating microglia at the CSA and in the adjacent striatum. (**B**) Microglial numbers at the CSA and striatum of E14.5 control and *Emx1*$^{cre/+}$;*Csf1*$^{fl/fl}$ mice ($n_{control}$ = 3 M; n_{mutant} = 3 F; from two distinct litters). (**C**) Microglial proliferation (EdU-positive) in the CSA and striatum of E14.5 control and *Emx1*$^{cre/+}$;*Csf1*$^{fl/fl}$ mice ($n_{control}$ = 3; n_{mutant} = 3; from two distinct litters). (**D**) Schematic representation of a hemicoronal section of an E14.5 mouse telencephalon showing the pattern of recombination driven by the *Emx1*^{cre} line in the embryonic brain, including progenitors and excitatory neurons of the neocortex. (**E**) Immunolabeling of coronal brain sections from E14.5 control and *Emx1*^{cre} embryos showing IBA1 and CD206-positive cells in the neocortex. (**F**) Microglial densities in the neocortex at E14.5 ($n_{control}$ = 3 (2 F;1 M), n_{cre} = 4 (1 F;3 M) from two distinct litters). (**G**) Schematic representation of a hemicoronal section of a E14.5 mouse telencephalon showing the pattern of recombination driven by the *Nestin*^{cre} line in the embryonic brain, including progenitors and excitatory neurons of the neocortex. (**H**) Immunolabeling of coronal brain sections from E14.5 control and *Nestin*^{cre};*Csf1*$^{fl/fl}$ embryos showing IBA1 and CD206-positive cells in the neocortex. (**I**) Microglial densities in the neocortex at E14.5 ($n_{control}$ = 3 (2 M;1 F), n_{homo} = 7 (4 M;3 F) from at least two distinct litters) (P = 0.0167). (**J**) Schematic representation of a hemicoronal section of an E14.5 mouse telencephalon showing the pattern of recombination driven by the *Emx1*^{cre} line in the embryonic brain, including progenitors and excitatory neurons of the neocortex. (**K**) Immunolabeling of coronal brain sections from E14.5 control and *Emx1*^{cre};*Il34*$^{fl/fl}$ embryos showing IBA1 and CD206-positive cells in the neocortex. (**L**) Microglial densities in the neocortex at E14.5 ($n_{control}$ = 7 (4 F;3 M), n_{homo} = 6 (6 F) from two distinct litters). Data were presented as mean ± SEM. Two-sided unpaired Mann–Whitney test were performed to assess differences (**B**, **C**, **F**, **I**, **L**). ns not significant, *$P < 0.05$. Scale bars: 500 µm (**A**, low mag); 100 µm (**A**, high mag), 200 µm (**E**, **H**, **K**). BAMs border-associated macrophages, CSA cortico-striatal-amygdalar boundary, CP cortical plate, IZ intermediate zone, SP subplate, Str striatum, SVZ subventricular zone, VZ ventricular zone.

 © The Author(s)

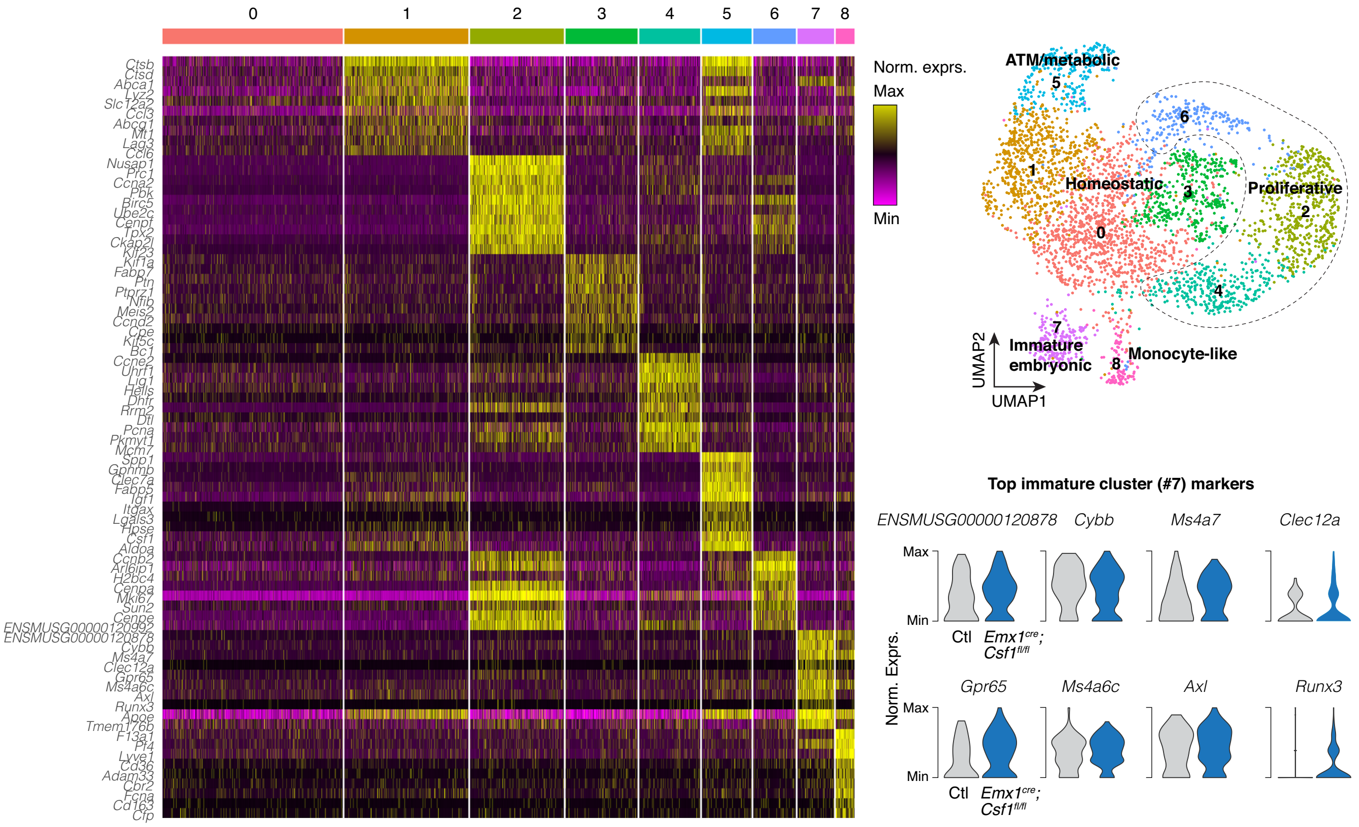

Figure EV4. Annotation and marker expression of microglia cluster.

Left, Expression of top ten markers (with avg_log2FC >1) for each microglial cluster across clusters. Right, Cluster annotation based on marker expression (top). Expression of top immature microglia markers in control and *Emx1*^{Cre}*;Csf1*$^{fl/fl}$ cluster 7 cells (bottom). Source data are available online for this figure.

© The Author(s)

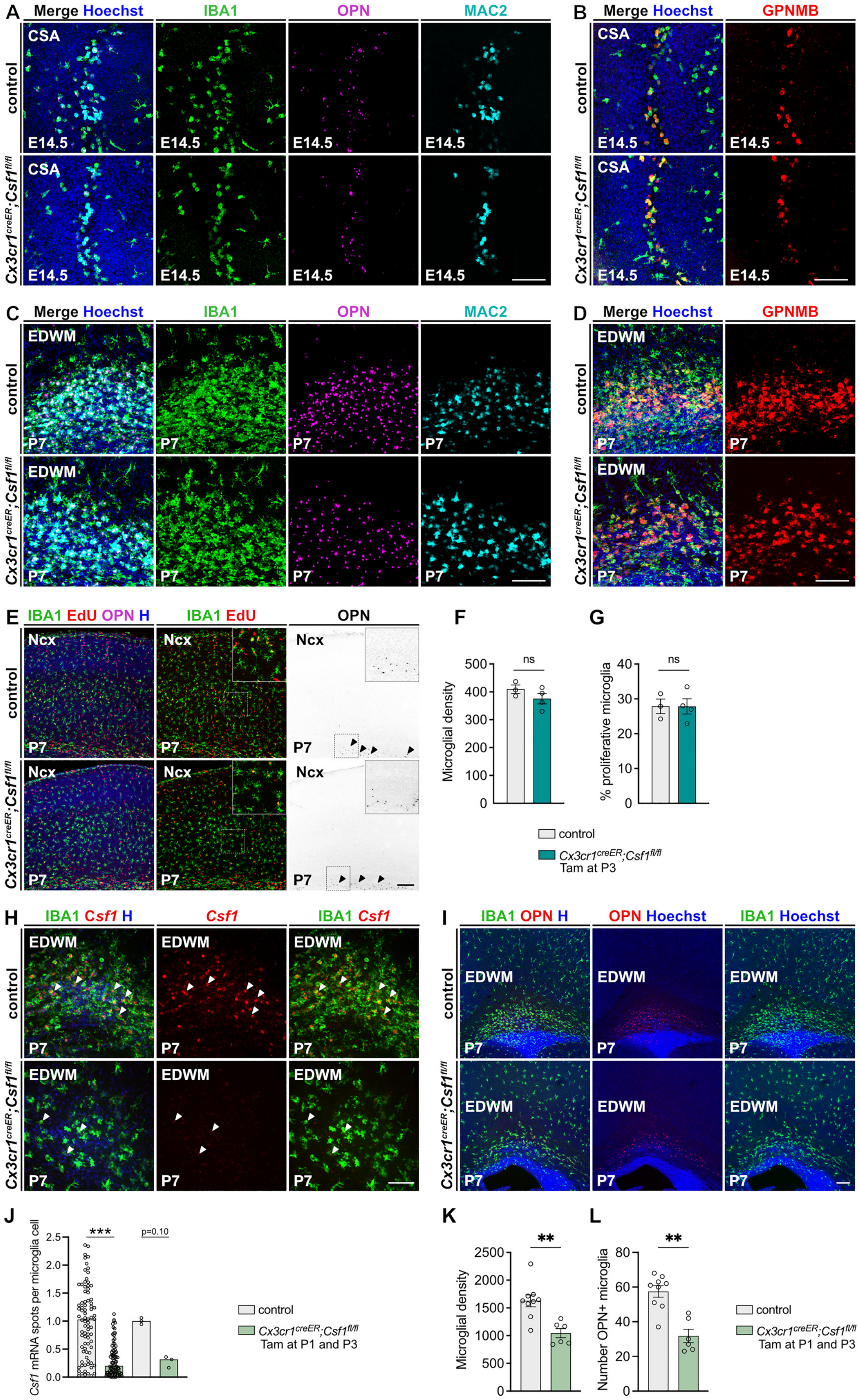
A
Merge Hoechst
IBA1
OPN
MAC2
B
Merge Hoechst
GPNMB
control
Cx3cr1creER;Csf1fl/fl
CSA
E14.5
C
D
EDWM
P7
E
IBA1 EdU OPN H
IBA1 EdU
OPN
Ncx
F
G
Microglial density
% proliferative microglia
ns
control
Cx3cr1creER;Csf1fl/fl
Tam at P3
H
IBA1 Csf1 H
Csf1
IBA1 Csf1
I
IBA1 OPN H
OPN Hoechst
IBA1 Hoechst
J
Csf1 mRNA spots per microglia cell
***
p=0.10
Tam at P1 and P3
K
L
Number OPN+ microglia
**

 © The Author(s)

Figure EV5. Microglial *Csf1* expression is specific to ATM accumulating in developmental hotspots and does not impact ATM core gene expression.

(**A**) Immunolabeling of coronal brain sections showing comparable co-expression of GFP, MAC2, and OPN at the CSA in E14.5 control and *Cx3cr1*$^{creER/+}$;*Csf1*$^{fl/fl}$ embryos (tamoxifen administered at E12.5) ($n_{control} = 5$; $n_{mutant} = 4$). (**B**) Immunolabeling of coronal brain sections showing comparable co-expression of GFP and GPNMB at the CSA in E14.5 control and *Cx3cr1*$^{creER/+}$;*Csf1*$^{fl/fl}$ embryos (tamoxifen administered at E12.5). (**C**) Immunolabeling of coronal brain sections showing comparable co-expression of GFP, MAC2 and OPN at the CSA in P7 control and *Cx3cr1*$^{creER/+}$;*Csf1*$^{fl/fl}$ embryos (tamoxifen administered at P3) ($n_{control} = 3$; $n_{mutant} = 3$). (**D**) Immunolabeling of coronal brain sections showing comparable co-expression of GFP and GPNMB at the CSA in P7 control and *Cx3cr1*$^{creER/+}$;*Csf1*$^{fl/fl}$ embryos (tamoxifen administered at P3). (**E**) Immunolabeling of coronal brain sections from control and *Cx3cr1*$^{creER/+}$;*Csf1*$^{fl/fl}$ mice showing absence of expression of OPN in cortical microglia (IBA1-positive) at P7 (tamoxifen administered at P3) while some white matter microglia do express OPN (solid arrowheads). Close-ups are 200 µm wide. (**F**) Proportion of proliferative microglia in control and *Cx3cr1*$^{creER/+}$;*Csf1*$^{fl/fl}$ neocortex of P7 mice (tamoxifen administered at P3)($n_{control} = 3$ (2 F;1 M) ; $n_{mutant} = 4$ (2 F;2 M); from one litter). (**G**) Microglial density (IBA1-positive cells/mm²) in control and *Cx3cr1*$^{creER/+}$;*Csf1*$^{fl/fl}$ neocortex of P7 mice (tamoxifen administered at P3)($n_{control} = 3$ (2 F;1 M) ; $n_{mutant} = 4$ (2 F;2 M); from one litter). (**H**) RNAscope experiments on coronal brain sections from P7 control and *Cx3cr1*$^{creER/+}$;*Csf1*$^{fl/fl}$ pups showing *Csf1* expression in IBA1-positive ameboid microglia at the CSA (solid arrowheads) after tamoxifen administration at P1 and P3. (**I**) Normalized quantification of RNAscope experiments on coronal brain sections from P7 control and *Cx3cr1*$^{creER/+}$;*Csf1*$^{fl/fl}$ embryos showing decreased *Csf1* expression in mutant mice. Left column shows all quantified cells and right column the mean expression per animal ($n_{control} = 3$ (1 F,2 M); $n_{mutant} = 3$ (2 M,1 F); from two distinct litters). (**J**) Immunolabeling of coronal brain sections from control and *Cx3cr1*$^{creER/+}$;*Csf1*$^{fl/fl}$ mice showing co-expression of IBA1 and OPN in ameboid microglia within the EDWM accumulation at P7 (tamoxifen administered at P1 and P3) ($n_{control} = 3$ (1 F,1 M) ; $n_{mutant} = 3$ (2 F;1 M); from one litter)(all cells, left: $P < 0.0001$; mean per animal, right: $P = 0.10$). (**K**) Microglial density (IBA1-positive cells/mm²) in control and *Cx3cr1*$^{creER/+}$;*Csf1*$^{fl/fl}$ EDWM of P7 mice (tamoxifen administered at P1 and P3) ($n_{control} = 9$ (3 F;6 M); $n_{mutant} = 6$ (3 F;3 M); from three distinct litters) ($P = 0.0028$). (**L**) Number of OPN-positive microglia at the EDWM at P7 ($n_{control} = 9$ (3 F;6 M); $n_{mutant} = 6$ (3 F;3 M); from three distinct litters) ($P = 0.0016$). Data were presented as mean ± SEM. Two-sided unpaired Mann–Whitney test were performed to assess differences (**F**, **G**, **J–L**). ns not significant, $^{**}P < 0.01$, $^{***}P < 0.001$. Scale bars: 100 µm (**A–D**, **H**, **I**); 200 µm (**E**). CSA cortico-striatal-amygdalar boundary, EDWM early-dorsal white matter, H Hoechst, Ncx neocortex.

© The Author(s)
